# *Laurus nobilis*, *Zingiber officinale* and *Anethum graveolens* Essential Oils: Composition, Antioxidant and Antibacterial Activities against Bacteria Isolated from Fish and Shellfish

**DOI:** 10.3390/molecules21101414

**Published:** 2016-10-22

**Authors:** Mejdi Snuossi, Najla Trabelsi, Sabrine Ben Taleb, Ameni Dehmeni, Guido Flamini, Vincenzo De Feo

**Affiliations:** 1 Laboratoire de Traitement et Valorisation des Rejets Hydriques (LR 15 CERTEO5), Technopole de Borj-Cédria, BP 273, Soliman 8020, Tunisie; snmejdi@yahoo.fr (M.S.); snmejdi@gmail.com (S.B.T.); dehmeni.ameni@yahoo.fr (A.D.); 2 Laboratoire de Biotechnologie de l’Olivier, Centre de Technologie de Borj-Cédria, BP 901, Hammam Lif 2050, Tunisie; najlatrabe@yahoo.fr; 3 Department of Pharmacy, University of Pisa, Via Bonanno 33, Pisa 56126, Italy; guido.flamini@farm.unipi.it; 4 Department of Pharmacy, University of Salerno, Via Giovanni Paolo II 132, Fisciano 84084, Salerno, Italy

**Keywords:** fish, shellfish, *Laurus nobilis*, *Zingiber officinale*, *Anethum graveolens*, essential oils, chemical composition, antioxidant activity, antibacterial activity

## Abstract

Several bacterial strains were isolated from wild and reared fish and shellfish. The identification of these strains showed the dominance of the *Aeromonas hydrophila* species in all seafood samples, followed by *Staphylococcus* spp., *Vibrio alginolyticus*, *Enterobacter cloacae*, *Klebsiella ornithinolytica*, *Klebsiella oxytoca* and *Serratia odorifera*. The isolates were studied for their ability to produce exoenzymes and biofilms. The chemical composition of the essential oils from *Laurus nobilis* leaves, *Zingiber officinale* rhizomes and *Anethum graveolens* aerial parts was studied by GC and GC/MS. The essential oils’ antioxidant and antibacterial activities against the isolated microorganisms were studied. Low concentrations of the three essential oils were needed to inhibit the growth of the selected bacteria and the lowest MBCs values were obtained for the laurel essential oil. The selected essential oils can be used as a good natural preservative in fish food due to their antioxidant and antibacterial activities.

## 1. Introduction

Fish and shellfish are excellent protein sources for human consumption due to their high content in vitamins, minerals and polyunsaturated fatty acids [1]. The organoleptic quality of marine products can deteriorate rapidly *post mortem* as a consequence of various microbial and biochemical breakdown processes. In fact, the degree of alteration depends on the initial bacterial count in the fresh fish and shellfish, which is also affected by the microbiological quality of the water, its temperature and salinity [2,3]. The bacterial flora in fish includes those naturally present in freshwater environments, and those associated with pollution of the aquatic environment. A third group includes bacteria introduced to fish and fish products during post-harvest handling and processing. In fact, procedures of handling, harvesting, processing (e.g., deheading, eviscerating, and cutting) and storage temperature are the main factors affecting the microbiological quality of fish and shellfish products *post-mortem* [4,5,6]. Seafood may be a vehicle for most known bacterial pathogens, such as *Salmonella*, *Edwardsiella tarda*, *Plesiomonas shigelloides*, motile *Aeromonas* and *Vibrio* spp. strains [7,8]. Filter feeders, such as mussels and oysters, concentrate all bacteria and viruses that live in the surrounding water in their filtration systems [8,9,10]. Recently, the 16S rDNA sequencing revealed *Shewanella baltica* as the main species isolated as hydrogen sulfide-producers from marine fish, followed by *Serratia* spp. and other *Shewanella* species [11].

Essential oils are aromatic and volatile oily liquids containing a mixture of compounds resulting from plant’s secondary metabolism formed in special cells found in leaves and stems, and commonly concentrated in one particular region such as leaves, bark or fruit. Some essential oils have shown promise as potential food safety interventions when added to processed and raw foods. Extracts/essential oils from dietary herbal species have been used as sources of medicine and food preservatives for over 4000 years [12]. Recently, some of the bioactive substances responsible for these medicinal and preservative activities have been identified as phenolic compounds like thymol and rosmarinic acid [13]. Many aromatic/medicinal spices and herbs are generally used in the Mediterranean basin to flavor fish and shellfish seafood preparations. These plants and their derivatives are also used as preservation agents to avoid bacterial seafood contamination [12,13,14]. Essential oils from oregano and mint are effective against the spoilage organism *Photobacterium phosphoreum*, even in fatty fish like codfish, and spreading the essential oil on the surface of whole fish or in a coating film for shrimps appears effective in inhibiting the respective natural spoilage flora [12]. The *Oreochromis niloticus* (L.) fry diet supplemented with ginger, black cumin, thyme, clove, watercress, fennel or garlic essential oils improves their fingerling performance [15,16,17]. The essential oils are recognized as safe according to the Food and Drug Administration [18]. In the food processing industry, additives and other ingredients are used during the technological process of production. Today, the most important trends in the food industry are the application of natural flavors, spices, antioxidants and pigments. Herbal drug extracts and essential oils are added in different kinds of food products as natural preservatives. Their basic performance is to improve the taste, smell and organoleptic effect on food production, digestion of food products, increase the freshness of products and at least preserve of the products in a proper way [12]. In Tunisia, zinger powder, dill aerial parts and dry laurel leaves are commonly added to fish and shellfish seafood dishes. Considering the great number of different chemical compounds groups present in the essential oils extracted from these plants, they are known to possess several biological activities including antibacterial, antifungal and antioxidant activities, and are used in various industries including medicine, food, and cosmetics [19,20].

The leaves of *L. nobilis* L. (*Lauraceae*) are usually used to treat gastrointestinal disorders, an in the cosmetics and food industry as a fragrance component. Laurel essential oil is generally dominated by the monoterpene compound 1,8-cineole. This species is used as a food flavouring agent, and in the pharmaceutical industry for drug formulations. Laurel essential oils are recognized for their antimicrobial activity against a wide panel of tested foodborne spoilage and pathogenic bacteria and fungi, antiviral and antibiofilm activities [21,22]. Commonly known as dill, *Anethum graveolens* (*Umbelliferae*) seeds are widely used in food and pharmaceutical industries to treat gastrointestinal problems (carminative, stomachic) and rheumatism. Carvone is the predominant odorant of dill seed and α-phellandrene, limonene, dill ether, myristicin are the most important odorants of dill herb. The essential oil of this annual aromatic herb is recognized to exhibited a wide range of biological activities including antibacterial, antifungal, antioxidant, anti-inflammatory, anti-hyperlipidemic and anti-hypercholesterolemic effects [23,24,25]. The plant *Zingiber officinale*, belonging to the family *Zingiberaceae* is indigenous to warm tropical climates, particularly southeastern Asia and is cultivated in India, China, Africa, Jamaica, Mexico and Hawaii. Both the essential oil and oleoresins extracted from the rhizomes (spice of commerce) are used in many food preparations, soft drinks and beverages [26]. The analysis of volatile oils from dill rhizome showed camphene, *p*-cineole, α-terpineol, zingiberene and pentadecanoic acid as major components. This oil possesses different biological properties including antibacterial, antifungal, ant-oxidative, anticancer, larvicidal, antidiabetic, anti-inflammatory and nephro/hepato-protective properties [27].

The main objectives of the present work were to study the antibacterial activities of *Laurus nobilis* (laurel), *Anethum graveolens* (dill) and *Zingiber officinale* (ginger) essential oils against bacteria isolated from fish and shellfish frequently consumed in Tunisia. The chemical composition and the antioxidant activities of the three essential oils were also studied.

## 2. Results

### 2.1. Identification and Characterization of Isolates

Seventy-threes trains were identified from all fish samples, mollusks and crustaceans analyzed based on biochemical characters obtained on the API galleries (20E, 20NE, Staph) and interpretation of results was performed by using the API software (bio Mérieux SA, Marcy l’Etoile, France). Ten species belonging to the genus *Staphylococcus* were identified in all samples tested: *S. lentus*, *S. xylosus*, *S. sciuri*, *S. lugdunensis*, *S. saprophyticus*, *S. epidermidis*, *S. aureus*, *S. hominis* and *S. cohnii* spp. *cohnii*, with dominance of the two species *S. lugdunensis* and *S. sciuri*. Only one strain isolated from the skin of sea bream was identified as *Micrococcus* spp.

**Figure 1:**
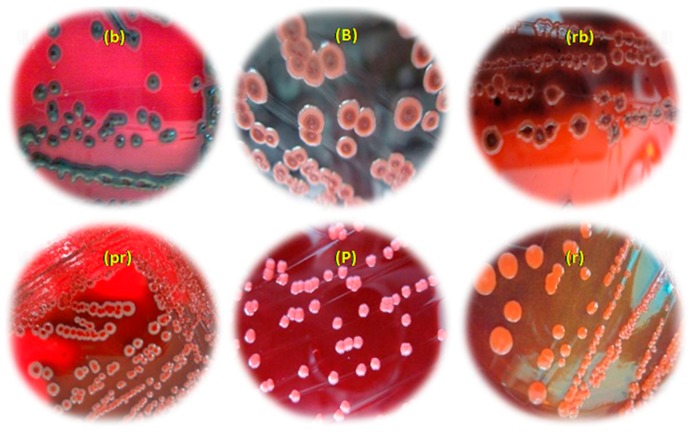
Morphotypes obtained on Congo red agar based on the colorimetric scale: (**b**): black colonies; (**B**): bordeaux colonies; (**rb**): red colonies with a darkening at the center; (**pr**): pink colonies with a red centre; (**P**): pink colonies; (**r**): red colonies.

Using the API20E galleries, the colonies growing on CHROMagar™ *E. coli* agar plates were identified as *Enterobacter cloacae*, *Klebsiella ornithinolytica*, *K. oxytoca* and *Serratia odorifera*. In addition, the 44 strains isolated from CHROMagar™ *Vibrio* agar plates and identified by using API20NE strips belonged to the genera *Aeromonas*, *Vibrio* and *Chromobacterium*. Only one strain isolated from the skin of sea bream (V_36_) has not been identified by the API 20NE system. *Aeromonas hydrophila* is the dominant species in all fish samples, mollusks and crustaceans analyzed. The strains selected in this study produced several hydrolytic enzymes such as amylase (100%), caseinase (100%), lecithinase (12/34: 35.29%) and DNase 24/34: 70.58%). The majority of strains were beta-haemolytic (24/34: 70.58%). All these data are summarized in Table 1. Using the Cristal Violet technique, all strains were interpreted as non-biofilm forming bacteria on polystyrene 96-well microtiter plate U-bottom as the optical densities estimated at 595 nm were less than 1. All tested strains grew on Congo red agar medium (Figure 1) giving five morphotypes with different color (red, pinkish-red, bordeaux, pink colonies with a darkening at the center, black). Among the isolated strains, three *A. hydrophila* and one *S. lentus* (11.76%) were slime producers characterized by black colonies, and the remaining 31 strains were non-slime producers.

### 2.2. Chemical Composition of the Essential Oils

The GC/MS analysis permitted the identification of 29, 24 and 60 components in the essential oils of laurel, dill, and ginger, respectively. Laurel essential oil is rich in monoterpene hydrocarbons (26.9%) and oxygenated monoterpenes (64.8%). The main constituents is this oil were 1,8-cineole (56%), α-terpinyl acetate (9.0%), 4-terpineol (5.2%), methyleugenol (3.6%), sabinene (3.5%) and α-pinene (3.2%). Carvone (27%), piperitone (25.7%), limonene (20.6%), dill apiol (8%), *trans*-dihydrocarvone (4.9%) and camphor (4.4%) were the key components in the essential oil of dill. Hydrocarbon monoterpenes (32%), oxygenated monoterpenes (31%) and hydrocarbon sesquiterpenes (22.2%) were the main chemical classes in ginger essential oil. This oil is rather rich in camphene (11.5%), β-phellandrene (10.7%), 1,8-cineole (10.4%), α-zingiberene (6.9%), borneol (6.4%), *ar*-curcumene (4.6%) and sesquiphellandrene (4.1%). The quali-quantitative compositions of the three oils are given in Table 2.

### 2.3. Antioxidant Activities

The assessment of antioxidant activity by a single method underestimates the antioxidant potential of an extract or molecule and it reflects only the ability to inhibit a precise class of oxidants present. The combination of different complementary assays can give a clearer idea of the antioxidant activity. For this, we evaluated in this study the antioxidant activity of the three essential oils using four antioxidant tests (DPPH assay, Ferrous Reducing power, capacities to inhibit the bleaching of β-carotene and to neutralize the superoxide anion).The results are reported in Table 3.

The DPPH assay is considered a valid and easy assay to evaluate the radical scavenging activity of antioxidants, since the radical compound is stable and does not have to be generated as in other radical scavenging assays.The scavenging effects on the DPPH radical expressed as IC_50_ values was the highest for *L. nobilis* essential oil (135 µg/mL) followed by *Z. officinalis* (470 µg/mL) and *A. graveolens* essential oils (3000 µg/mL), showing a radical scavenging activity clearly less important than that the positive control BHT (IC_50_ 11.5 µg/mL).

The inhibition of β-carotene bleaching is another type of antioxidant assay associated with lipid peroxidation. In oil–water emulsion-based system, linoleic acid undergoes thermally induced oxidation, thereby producing free radicals that attack the β-carotene chromophore resulting in a bleaching effect. The IC_50_ value registered in the β-carotene bleaching assay was lower for *Z. officinalis* (1900 mg/mL) followed by *L. nobilis* (3600 mg/mL) and *A. graveolens* (3000 mg/mL) essential oils. With regards to the superoxide radical anion assay, our results indicated a high scavenging ability for the ginger essential oil (IC_50_ 320 µg/mL) as compared to the laurel and dill essential oils, although lower than the standard, BHT (IC_50_ 1.5 µg/mL).

As shown in Table 3, the reducing power of the three essential oils, expressed as EC_50_, was clearly less important than that of positive control, ascorbic acid (37.3 µg/mL), and the highest activity was registered for *L. nobilis* essential oil (1850 mg/mL), followed by *Z. officinalis* essential oil (1900 mg/mL) and *A. graveolens* (1900 mg/mL).

### 2.4. Antibacterial Activities

The antimicrobial activities of the three essential oils against the microorganisms isolated from seafoods products were qualitatively and quantitatively evaluated by the presence or the absence of inhibition zone diameter, MICs and MBCs values. The results were given in Table 4.

Based on the observed means compared using the Duncan’s multiple range tests for means, three groups were observed and *L. nobilis* essential oil showed the highest activity, regardless of the bacteria tested (mean = 14.25 mm) followed by *A. graveolens* (mean = 12.31 mm) and *Z. officinale* essential oils (mean = 11.36 mm). The results showed that the three essential oils had substantial antimicrobial activity against the 34 bacteria tested as compared to the results obtained with the standard compounds erythromycin and kanamycin. In fact, the data obtained in terms of zones of growth inhibition (mm) scored in Mueller-Hinton agar demonstrated that *Z. officinale* essential oil produced the highest diameters of growth inhibition (between 6–22.33 mm). This oil was active against all Gram negative bacteria, especially *A. hydrophila* with a diameter of inhibition ranging from 10.33 to 22.33 mm and the Gram negative *Staphylococcus* spp. with a diameter of inhibition ranging from 6 to 15.33 mm. The highest diameter of inhibition (about 25.33) mm was obtained when *A. graveolens* essential oil was tested against the *A. hydrophila* (R5) isolated from the blue mussels *Mytilus galloprovincialis*.

The MIC and MBC values indicate that the tested essential oils were active against *A. hydrophila*, *Enterococcus* spp., *Klebsiella* spp., *Staphylococcus* spp., *S. odorifera* and *V. alginolyticus* strains, with low MICs values ranging from 0.05 to 0.39 mg/mL, while high concentrations of essential oils were needed to inhibit growth of almost all isolated bacteria. The lowest MBCs values were obtained with the laurel oil and the concentrations needed to inhibit the growth of *A. hydrophila* strains ranged from 6.25 to 50 mg/mL.

## 3. Discussion

### 3.1. Identification and Characterization of Isolates

The results of the identification of bacterial strains isolated from fish and shellfish sampled obtained in this study agree with those previously reported by Boulares and co-workers [10] who described *Pseudomonas fluorescens*, *P. putida*, *A. hydrophila*, and *Photobacterium damselae* as the dominant psychrophic species isolated from both wild and reared fish in Tunisia. In fact, the majority of the isolated psychrophic strains were able to produce several exoenzymes (gelatinase, protease, lipase, and lecithinase). Additionally, several works reported the isolation of *Vibrio* spp. and *A. hydrophila* strains from reared fish (*D. labrax*, *S. aurata*) and mollusks (*M. galloprovincialis*, *Tapes decussatus* and *Crassostrea virginica*) able to produce several exoenzymes and multiresistant to several antibiotics [28]. Recently, Saidi and co-workers [29] used the API 20 NE galleries to identify strains of *A. hydrophila* from Tunisian aquatic biotopes and from ornamental fish which were able to produce several exoenzymes including lipase, caseinase, amylase, proteases and lecithinase. Additionally, other Khouadja and colleagues [30] reported the isolation of *P*. *damselae* subsp. *damselae* from different outbreaks affecting cultured *S. aurata* and *D. labrax*. The edible lamellibranches mollusks are frequently the cause of very serious outbreaks of food poisoning due the presence of several pathogenic bacteria like the two fecal contaminants *E. coli* and *Salmonella* sp., and especially members of the *Vibrionaceae* family [8].

*Staphylococcus* spp. species are also one of the most important food borne opportunistic bacteria isolated from fish samples and the high population of these bacteria indicates the general quality of fish. In the present work, most isolated *Staphylococcus* spp. strains (*S. saprophyticus*, *S. epidermidis*, *S. hyicus*, *S. aureus* and *S. intermedius*) were previously reported as the main bacteria isolated from the skin, muscle and internal organs of both *Cyprinus carpio* and *Silurus glanis* fishes [31].

In previous works, we reported the isolation and characterization of *Vibrio* spp. strains able to use the fish mucous as a sole source of carbon and to produce a biofilm on several abiotic surfaces. These properties may allow them to persist in the aquatic biotope in a free-living planktonic state or attached to biotic and abiotic surfaces [32,33]. Sechi and co-workers [34] studied the virulence properties of *Aeromonas* spp. isolated from coastal water and clinical sources in Sardinia (Italy) and reported a high adhesion capacity among strains isolated from patients with diarrhoea. In fact, 35.3% of environmental *A. hydrophila* tested were positive in slime test characterized by black colonies [34]. Saidi and co-workers [29] studied the adhesive properties of *A. hydrophila* strains isolated from ornamental fish and reported that the majority of strains were able to adhere to skin mucus, and cells (Hep-2 and Caco-2). In addition, on Congo Red Agar, only the 65% of *A. hydrophila* strains produced slime, the 65% of the strains were able to form biofilm on glass tube with Crystal violet and the 85% with Safranin. Most *A. hydrophila* strains (95%) were adhesive to polystyrene with high optical density values. More recently, Thenmozhi and co-workers reported that 76.2% of *A. hydrophila* strains isolated from carp fish produced pigmented colonies (black) on Congo Red Agar plates [35]. For the *V. alginolyticus* species, we characterized several morphotypes with different colour (red, pinkish-red, black) isolated from *S. aurata*, *D. labrax* and from aquatic biotopes [32,33]. The strains were also able to form biofilm on several abiotic surfaces including those commonly used in fish aquaculture installations (glass, polystyrene, polyethylene and polyvinyl-chloride). Ben Abdallah and co-workers reported that the food-borne pathogen *V. alginolyticus* isolated from the internal organs of aquacultured diseased gilthead sea bream (*S. aurata*) and sea bass (*D. labrax*) gave white colonies on CRA plate [36]. For the *Staphylococcus* spp. species, special attention was focused on clinical *S. aureus* one. Few studies have reported the virulence properties including biofilm formation by the *Staphylococcus* coagulase- negative strains isolated from seafood’s products. Vázquez-Sánchez and co-workers reported the isolation of *S. aureus* strains from seafood marketed in Galicia (Northwest Spain) capable of developing biofilms on food-processing surfaces [37].

### 3.2. Chemical Composition of the Essential Oils

The studied chemotype of laurel essential oil harvested from Tunisian was similar to those previously observed from Morocco, Turkey, Algeria and Tunisia, Argentina and Italy with high content of 1,8-cineole [38]. In fact, our results agree with those reported by Marzouki and co-workers [39] for laurel essential oil from Tunisia and Algeria who presented as major components 1,8-cineole, linalool, α-terpinyl acetate, methyl eugenol, sabinene and (*E*)-caryophyllene. Recently, Ben Jemâa and co-workers [38] reported that 1,8-cineole, linalool and isovaleraldehyde were the common constituents in Algerian, Moroccan and Tunisian laurel essential oil. These variations in the main constituents reported in the literature can be explained by the influence of environmental and methodological factors [40]. Additionally, for the dill essential oil, our results agree with those reported by Peerakam and co-workers [41] who founded that monoterpene hydrocarbons (38.83%), oxygenated monoterpenes (38.83%) and aromatic ethers (16.67%) were the major component groups in the essential oil of *A. graveolens* seeds from Thailand. This oil was particularly rich in d-carvone (32.94%), carvone (20.73%), dill apiol (19.64%) and limonene (18.08%). Essential oils from other countries are reported to possess similar chemical pattern, but differences in the relative quantity of chemical compounds in the EO. In fact, carvone (45.9%–75.2%) and limonene (18.4%–21.6%) were described as major components in dill essential oil from Romania and Estonia [42] and from Bulgaria [43]. Bailer and co-workers [44] indicated carvone and limonene as the major constituents of dill seed oils, and α-phellandrene is the main constituent of dill weed oil. The differences in the chemical profiles can be also attributed to plant cultivation, growth stage, climatic conditions, cultivar, plant density, nutrient application, extraction methods [45].

In the literature, many variations have been found in the chemical composition of rhizome ginger oil. In fact, curcumene was reported as the main constituents in the fresh ginger rhizomes [46], while Menut and co-workers [47] identified citral as the main constituent of ginger oil. Additionally, geranial (25.9%), α-zingiberene (9.5%), (*E,E*)-α-farnesene (7.6%), neral (7.6%) and ar-curcumene (6.6%) were identified as the main components in the rhizomes essential oil from Gorakhpur, India [41], with α-zingiberene as the main component [48,49]. Sivasothy and colleagues [50] studied the chemical composition of *Z*. *officinale* var. *rubrum Theilade* essential oils and reported the isolation of 54 components from the rhizomes and 46 from the leafs. The leaf essential oil was particularly rich on β-caryophyllene (31.7%), while camphene (14.5%), geranial (14.3%) and geranyl acetate (13.7%) were the three dominant compounds in the oil from the rhizome. Yeh and co-workers [51] identified the bioactive components of two varieties of zinger rhizomes (GG: Guangdong-ginger and CG: Chu-ginger) from Mingjian (Taiwan) and reported that gingerols, shogaol and curcumin as major compounds. The two varieties exhibited similar volatile profiles and 60 and 65 compounds were identified for GG and CG, respectively. Monoterpenoids, sesquiterpenoids and aldehydes were the major classes of compounds in the two essential oils. Additionally, eight major components were identified as camphene (6.27% and 9.14%), sabinene (8.16% and 10.67%), α-curcumene (8.42% and 6.66%), zingiberene (20.85% and 17.55%), α-farnesene (7.42% and 4.77%), β-sesquiphellandrene (8.13% and 7.32%), neral (8% and 6.74%), and geranial (8.7% and 7.2%) for GG and CG, respectively. The differences in the chemical profiles could be due to environmental, developmental and genetic factors, production conditions, variety, cultivars or population and climatic and soil factors [49,50,51].

### 3.3. Antioxidant Activities

Our results showed that laurel essential oil possessed effective antioxidant activity using the DPPH and β-carotene bleaching techniques, which can be attributed to the presence of phenolic compounds [50,51]. Cherrat and co-workers [52] reported that an essential oil from Moroccan laurel possessed effective DPPH scavenging activity at low concentrations (1.25 µL/mL), strong reductive potential which increase with increasing concentrations (in the range of 2.5–10 µL/mL) and was also able to inhibit linoleic acid oxidation reaching an inhibition of 53.3% at 1.25 µL/mL and 90.7% at 10 µL/mL. Recently, Basak and co-workers [53] reported that a Turkish laurel essential oil possessed DPPH, hydroxyl, superoxide radical and hydrogen peroxide scavenging activities greater than the controls (curcumin, ascorbic acid, BHT).

Many researchers reported the antioxidant activities of dill essential oils [54,55]. In fact, Peerakam and co-workers [41] showed that the dill essential oil from Thailand possessed high total phenolic content (4.5746 mg GAE/mL) and higher antioxidant activities on DPPH, followed by ABTS and FRAP assays (Trolox equivalence antioxidant capacity = 52.5391 mg/mL, 1.5936 mg/mL, 0.5469 mg/mL; respectively). These activities can be attributed to the high content in monoterpene hydrocarbons and oxygenated monoterpenes in the essential oil [54]. Additionally, ginger is one of the most widely used herbs that contains several interesting bioactive constituents and possesses health promoting properties [55,56,57]. Previous results have shown the antioxidant activities of ginger essential oil and their bioactive components. In fact, Rehman and co-workers [58] found that ginger essential oil possess good thermal stability and exhibited 85.2% inhibition of peroxidation of linoleic acid when heated at 185 °C for 120 min, and ABTS scavenging capacity (989.7 ± 44.2 µmol·trolox/g). Therefore the use of ginger essential oil is recommended as a natural antioxidant to suppress lipid oxidation in processed food [58].

Such antioxidant activities can inhibit in various ways the interaction of free radicals in the form of ROS or NOS with various biomolecules (proteins, amino acids, lipids and DNA), and lead to decreased cell death and damage [59].

### 3.4. Antibacterial Activities

Previous results reported the effectiveness of laurel, dill and ginger essential oils from different origins against a wide panel of Gram positive and Gram negative tested foodborne spoilage and pathogenic bacteria [60,61,62,63,64,65,66]. In fact, laurel essential oil was active against *S. aureus*, *S. epidermidis*, *E. faecalis* and *P. aeruginosa*, *S. aureus*, *S. intermedius* and *K. pneumoniae*, *E. coli* O157:H7, *L. monocytogenes* and *S.* Typhimurium. It was shown, that dill oil exhibited antimicrobial activity against *S. aureus*, *E. coli*, *C. albicans* and *A. flavus* strains with a diameter of inhibition ranging from 16 to 30 mm. Indeed, Lopez and co-workers [62] reported the antimicrobial activity of dill essential oil against foodborne bacterial and fungal strains including four Gram positive bacteria (*S. aureus*, *B. cereus*, *E. faecalis*, and *L. monocytogenes*), four Gram negative bacteria (*E. coli*, *Y. enterocolitica*, *S. choleraesuis*, and *P. aeruginosa*), and three fungi (a yeast, *C. albicans*, and two molds, *P. islandicum* and *A. flavus*). In many ginger samples, several bioactive compounds (gingerols and shogaols) with antibacterial activity have been found [64,65]. The ginger essential oil possess inhibitory effects against *S. aureus*, *L. monocytogenes*, *P. aeruginosa*, *S. Typhimurium*, *Shigella flexineri* and *E. coli*, with a diameter of inhibition zone ranging from 1.16 to 8.66 mm [66].

Using the scheme proposed for the classification of the essential oils by Aligiannis and co-workers [67] (strong activity is for MIC values between 0.05–0.5 mg/mL, moderate activity MIC values between 0.6–1.5 mg/mL and weak activity above 1.5 mg/mL), it appear that the three tested essential oils possessed strong activity against the different microorganisms tested as the MICs values for all volatile oils ranged from 0.05 to 0.2 mg/mL for zinger and laurel oils and from 0.05 to 0.39 mg/mL for the dill oil. In the MIC/MBC assays, laurel essential oil presented strong to moderate activity (MIC ≤ 5 mg·mL^−1^) against *Lactobacillus plantarum, L. monocytogenes, E. faecalis, B. cereus, B. subtilis, E. coli, S.* Typhimurium, *P. vulgaris, E. aerogenes, P. aeruginosa* and *Y. enterocolitica,* except for *S. aureus* [67]. In 2008, Erkmen and Özcan [68] evaluated the antimicrobial activity of a laurel essential oil, containing approximately 60% 1,8-cineole and only traces of linalool, and reported a MIC of 0.02% and cidal affect at of 0.2% against *S. aureus*, *E. faecalis*, and *L. monocytogenes*. The MIC and BC (bactericidal concentration) were 0.2% and 1.0% against *B. cereus* and *B. subtilis*, respectively. The same authors reported that this oil was ineffective against *E. coli* and *S.* Typhimurium while bactericidal against *Y. enterocolitica* at 2.5%. For Tunisian laurel oil, Bouzouita and colleagues [69] reported that laurel oil (42.3% 1,8-cineole, 25% linalool and 11.2% α-terpinyl acetate) exhibited antimicrobial activities against *E. coli* with a final cell concentration ranging from 10^11^ to < 10^2^ UFC/mL at 2.5% (*v/v*). Recently, Merghni and co-workers [19] reported that Tunisian laurel essential oils harvested from Sousse locality (Center of Tunisia) displayed varying degree of activities against oral Staphylococci with MIC values ranging from 3.91 to 62.50 mg/mL.

For the *Z*. *officinale* oil, Hamad and colleagues [70] reported that the MICs values of Indonesian ginger oil evaluated by broth dilution method against foodborne bacteria *B. subtilis*, *E. coli*, *S. aureus*, *S*. Typhimurium and *V. cholera* were ranging from 0.5 to 1 mg/mL. Recently, Sivasothy and colleagues [50] reported that both leaf and rhizome essential oils from *Z*. *officinale* var. *rubrum Theilade* were moderately active against the Gram positive bacteria *Bacillus licheniformis*, *B. spizizenii* and *S. aureus*, and the Gram negative bacteria *E. coli*, *K. pneumoniae* and *P. stutzeri*, with MICs values ranging from 0.16–0.63 mg/mL. It was also reported that compounds such as α-pinene, α-terpineol and geraniol, present at low concentrations in the zinger essential oil, affected the overall efficiency of the antibacterial activity of the oil, indicating a synergistic effect of all components. Using the incorporation method, Bellik and co-workers reported that the MICs values of ginger essential oil ranged from 8.69 mg/mL for *S. aureus*, 86.92 mg/mL for *B. subtilis* and 173.84 mg/mL for *E. coli* strains [57].

The dill essential oil was also active against *S. aureus*, *E. coli*, *C. albicans* and *A. flavus* strains with a MICs values ranging from 5.99 to 59.47 mg/mL [41]. In addition, Peerakam and co-workers [41] founded that the essential oil from the aerial parts of *A. graveolens* showed strong inhibition at the lowest concentration (2.66 µg/mL) against *S. aureus*, and (85.77 µg/mL) against *E. coli.* Previous researches explained the antimicrobial activity of dill essential oil by the presence of components, like d-carvone and limonene characterized by a strong antifungal activity [40]. In fact, Ruangamnart and co-workers [71] reported that d-limonene and d-carvone exhibited antibacterial activity against Methicillin Resistant *S. aureus* ATCC 43300 (MRSA) with MIC values of 0.3125 and 1.25 mg/mL, respectively and MBC values of 1.25 and 2.5 mg/mL, respectively. Strains of *S. aureus* ATCC 25923, *S. sobrinus* ATCC 33478, *B. subtilis* ATCC 6633, *E. coli* ATCC 8739 and *S.* Typhimurium ATCC 14028 were more sensitive to d-limonene than d-carvone and dill oils with the MIC values of 1.25–5 mg/mL and MBC values of 1.25-10 mg/mL, whereas, *S. aureus* ATCC 6538, *S. aureus* ATCC 29213 and *K. pneumoniae* ATCC 700603 had quite the same MIC and MBC values with rang 5-10 mg/mL in both d-limonene and d-carvone. *P. aeruginosa* ATCC 27853 and *P. aeruginosa* ATCC 9027 were not killed at 10 mg/mL of essential oil [72].

## 4. Materials and Methods

### 4.1. Sampling and Strains Isolation

Foodborne strains, used in this work, were isolated from aquacultured fresh fish (*D. labrax*: sea bass, *S. aurata*: sea bream) and from shellfish (*Parapenaeus longirostris*: rose shrimp, *Mytilus galloprovincialis*: blue mussel). These samples were obtained from a local market (Souk El Blat, Tunisia) packed in cold boxes and transferred to the laboratory within 2 h for analyses [2]. Upon arrival, fish were immediately gutted, headed, washed and filleted. Mussels were washed, scrubbed free of dirt, shucked with a sterile knife. Twenty-five grams of meat and liquor of blue mussels muscle, and from each fish species were transferred aseptically and diluted in Stomacher packet each containing 225 mL of alkaline peptone water supplemented with 1% NaCl. The mixture was homogenized for 3 min using a laboratory blender Stomacher then incubated at 37 °C for 18–24 h. The enrichments (100 μL) were then streaked onto Thiosulfate-citrate-bile salt-sucrose agar (TCBS agar, Tulip Diagnostics. (P) LTD, Verna Industrial Estate, India), CHROMagar™ *Vibrio* and CHROMagar™ *S. aureus* media (*C*HROMagar Microbiology, Paris, France) and incubated for 18–24 and 37 °C.

### 4.2. Identification and Biochemical Characterization of Bacterial Strains

Bacterial strains growing on the selective mediums plates (Green and yellow colonies growing on TCBS plates, mauve, green blue/turquoise blue and white colonies from CHROMagar™ *Vibrio* plates) were purified on Tryptic soy agar plates supplemented with 1% NaCl (HiMedia Laboratories Pvt, Ltd., Mumbai, India), while the pink to mauve colonies on CHROMagar™ *S. aureus* medium were purified on Tryptic soy agar. All pure colonies were subjected to standard morphological, physiological, biochemical plate and tube tests [73]. A commercial miniaturized Api 20E and Api 20NE systems (Bio Mérieux SA, Marcy l’Etoile, France) was used to identify all the strains isolated from TCBS and CHROMagar™ *Vibrio* media. The Api Staph strips (Bio Mérieux) were used to identify the bacterial strains isolated from the CHROMagar™ *S. aureus* plates.

For Exoenzymes production, all strains were tested for haemolysin (Human blood agar, Pronadisa Laboratories Coda, S.A) and DNA hydrolysis (DNase Agar, Biolife, S.r.l., Milano, Italy). The enzymes amylase, caseinase and lecithinase were detected on media prepared with Phosphate Buffer Saline (PBS) supplemented with 0.5% casein peptone, 5% skim milk powder and 5% egg yolk emulsion, respectively [32].

### 4.3. Adhesive Properties of the Selected Strains

The adhesive properties of the selected strains were tested qualitatively using the Congo Red technique and quantitatively by using the Crystal Violet technique [33].

For the Congo Red medium was prepared by adding 0.8 g/L Congo red (QualiKems Fine Chem Pvt. Ltd., Nandesari, Vadodara, India) and 36 g of Saccharose (Labosi, France), both of which had been previously autoclaved separately, to 1 L of Brain Heart Infusion Agar (Scharlau Microbiology, Pronadisa, Madrid, Spain). The Congo Red stain was prepared as a concentrated aqueous solution and autoclaved separately at 121 °C for 15 min and was added when the agar had cooled to 55 °C. Plates were incubated at room temperature at 37 °C for 24 h under aerobic conditions and followed overnight at room temperature. After incubation, pigmented colonies (generally black colour) were considered as slime positive, whereas unpigmented bacteria (formed pinkish red, smooth colonies with a darkening at the centre) were interpreted as slime-negative strains.

The ability of the identified 34 strains to form a biofilm on abiotic surface was quantified using the protocol described by Toledo-Arana and co-workers [74]. Al strains were grown overnight in Brain Infusion Broth (BHI-0.25 glucose at 37 °C). The culture was diluted 1:20 in fresh BHI plus (0.25%) glucose at 37 °C. 200 µL of this suspension was used to inoculate sterile 96-well-polystyrene microtiter plates (Nunc, Roskilde, Denmark). The plates were incubated aerobically at 37 °C for 24 h. The cultures were removed and the microtiter wells were washed twice with phosphate-buffered saline (7 mM Na_2_HPO_4_, 3 mM NaH_2_PO and 130 mM NaCl at pH 7.4) to remove non-adherent cells and were dried in an inverted position. Adherent bacteria were stained with 1% Crystal violet (Merck, Paris, France) for 15 min. The wells were rinsed once more and the Crystal Violet was solubilised in 200 µL of ethanol-acetone (80:20 *v/v*). The optical density (OD_595nm_) was measured in spectrophotometer (Amadèo Bibby, Sterling, France). The following values were assigned for biofilm determination: non biofilm forming OD_595_ = 1; weak biofilm forming 1 < OD = 2; medium biofilm forming 2 < OD = 3; strong biofilm forming OD_595_ = 3. Each essay was performed in triplicate.

### 4.4. Plant Material, Extraction and Chemical Characterization of the Essential Oil

*A*. *graveolens* seeds and *Z. officinale* (rhizome) were purchased from a local market in Tunisia (Souk El Blat). The *L. nobilis* leaves were collected from trees growing wild in Grombalia (Tunisia). The plants were identified by Pr. Abderrazak Smaoui and voucher specimens are preserved in the Laboratoire de Traitement et Valorisation des Rejets Hydriques (LR 15 CERTE O5, Technopole de Borj-Cédria, Tunisia). One hundred grams of fresh material was subjected to hydrodistillation during three hours using 500 mL of distilled water in a Clevenger-type apparatus [75]. The oil obtained was collected and dried over anhydrous sodium sulfate and stored in sealed glass vials in a refrigerator at 4 °C.

GC/EIMS analyses were performed with a CP-3800 gas-chromatograph (Varian, Palo Alto, CA, USA) equipped with a HP-5 capillary column (30 m × 0.25 mm; coating thickness 0.25 μm) and a Varian Saturn 2000 ion trap mass detector. Analytical conditions: injector and transfer line temperatures at 220 and 240 °C, respectively; oven temperature was programmed from 60 to 240 °C at 3° C/min; carrier gas helium at 1 mL/min; injection of 0.2 µL (10% hexane solution); split ratio 1:30. Most constituents were identified by gas chromatography by comparison of their Kovats retention indices (R_i_) (determined relative to the t_R_ of *n*-alkanes (C_10_–C_35_)), with either those of the literature [76,77,78,79,80] and mass spectra on both columns with those of authentic compounds available in our laboratories by means of NIST 02 and Wiley 275 libraries [75]. The components’ relative concentrations were obtained by peak area normalization. No response factors were calculated.

### 4.5. Antioxidant Properties

#### 4.5.1. DPPH Radical-Scavenging Activity

The antioxidant activity of the three essential oils was measured in term of hydrogen donating or radical scavenging ability using the stable DPPH method [81] Briefly, 0.25 mL of a 0.2 mM DPPH methanolic solution was mixed with 1 mL of essential oil extract at different concentrations (50 to 2000 µg/mL). The mixture was left for 30 min at room temperature in the dark. The absorbance of the resulting solution was then read at 517 nm. The antiradical activity was expressed as IC_50_ (µg·mL^−1^). The ability to scavenge the DPPH radical was calculated using the following Equation (1):

DPPH scavenging effect (%) = [(A_0_ − A_1_)/A_0_] × 100 (1)

where A_c_ is the absorbance of the control at 30 min, and A_s_ is the absorbance of the sample or control (BHT at 1, 10, 20 and 50 µg/mL) at 30 min. The essential oil and BHT concentrations (µg/mL) providing 50% of DPPH radical scavenging activity (IC_50_) were calculated. All samples were analyzed in triplicate.

#### 4.5.2. Superoxide Anion Scavenging Activity

Superoxide anion scavenging activity was assessed using the method described by Duh and coworkers [82]. The reaction mixture contained 0.2 mL of essential oil at different concentrations (200 to 2000 µg/mL), 0.2 mL of 60 mM PMS stock solution, 0.2 mL of 677 mM NADH, and 0.2 mL of 144 mM NBT, all in phosphate buffer (0.1 mol/L, pH 7.4). After incubation at ambient temperature for 5 min, the absorbance was read at 560 nm against a blank. Evaluating the antioxidant activity was based on IC_50_. The IC_50_ index value was defined as the amount of antioxidant necessary to reduce the generation of superoxide radical anions by 50%. The IC_50_ values (three replicates per treatment) were expressed as μg/mL. As for DPPH, a lower IC_50_ value corresponds to a higher antioxidant activity of plant extract. The inhibition percentage of superoxide anion generation was calculated using the following formula Equation (2):

Superoxide quenching (%) = [(A_0_ − A_1_) × 100]/A_0_ (2)

where A_0_ and A_1_ have the same meaning as in Equation (1).

#### 4.5.3. Determination of Reducing Power

The ability of the essential oils to reduce Fe^3+^ was assayed by the method of Oyaizu [83]. Briefly, 1 mL of essential oil was mixed with 2.5 mL of phosphate buffer (0.2 mol/L, pH 6.6) and 2.5 mL of K_3_Fe(CN)_6_ (1 g/100 mL). After incubation at 50 °C for 25 min, 2.5 mL of trichloroacetic acid (10 g/100 mL) was added and the mixture was centrifuged at 650 × *g* for 10 min. Finally, 2.5 mL of the upper layer was mixed with 2.5 mL of distilled water and 0.5 mL of aqueous FeCl_3_ (0.1 g/100 mL). The absorbance was measured at 700 nm. The mean of absorbance values were plotted against concentration and a linear regression analysis was carried out. Increased absorbance of the reaction mixture indicated increased reducing power. EC_50_ value (mg·mL^−1^) is the effective concentration at which the absorbance was 0.5 for reducing power. Ascorbic acid was used as positive control.

#### 4.5.4. β-Carotene-linoleic Acid Model System

The anti-peroxyl radical activity was evaluated by measuring the peroxides generated during the oxidation of linoleic acid at high temperature according to the method of Hajlaoui and co-workers [84] with some modifications. Briefly, 0.2 mg of β-carotene was dissolved in 2 mL of chloroform, and added with 20 mg of linoleic acid and 200 mg of Tween 40. After removing CHCl_3_ under vacuum, oxygenated water (100 mL) was added, and the flask was vigorously shaken until all material dissolved. The emulsion obtained was freshly prepared before each experiment. An aliquot of 150 μL of emulsion was distributed in each of the wells of 96-well microtiter-plates and 10 mg of the EO or BHA standard solution was added. An equal amount of emulsion was used for the blank sample. The microtiter plate was incubated at 45 °C and the absorbances were measured at 490 nm using a visible/UV microplate kinetics reader (EL × 808, Bio-Tek instruments, Winooski, VT, USA). Readings of all samples were performed immediately (t = 0 min) and after 120 min of incubation. The antioxidant activity (AA) of the extracts was evaluated in terms of β-carotene blanching using the following formula Equation (3):

AA% = [(A_0_ − A_t_)/A_0_] × 100 (3)

where A_0_ is the absorbance of the control at 0 min, and A_t_ is the absorbance of the sample at 120 min. The results are expressed as IC_50_ values (μg/mL).

### 4.6. Antimicrobial Activities

#### 4.6.1. Microorganisms

Thirty four strains isolated from aquacultured fresh fish (*D. labrax*: sea bass, *S. aurata*: sea bream) and shellfish (*P. longirostris*: rose shrimp, *M. galloprovincialis*: blue mussel) were selected to test the in vitro antibacterial activities of the three essential oils.

#### 4.6.2. Disk-Diffusion Assay

The antimicrobial activity testing was done according to the protocol described by Snoussi and co-workers [85]. For the experiments, a loopful of the microorganism working stock was enriched in a tube containing 9 mL of enrichment broth (MH) then incubated at 37 °C for 18–24 h. The overnight cultures were used for the antimicrobial activity of the essential oils used in this study and the optical density was adjusted to 0.1 standard turbidity (OD_600nm_). The inoculums were streaked onto MH agar (1% NaCl) plates using a sterile swab. Sterile filter discs (diameter 6 mm, Biolife, Milano, Italy) were impregnated with 10 mg of essential oil. Erythromycin and kanamycin were used as positive controls for all bacterial strains tested. The antibiotic susceptibility was determined by using the Kirby-Bauer method [86]. The plates were incubated at 37 °C for 18–24 h. The diameter of the zones of inhibition around each of the disks was taken as measure of the antimicrobial activity.

#### 4.6.3. Microdilution Method for the Determination of the MICs and MBCs

The minimal inhibition concentration (MIC) and the minimal bactericidal concentration (MBC) values were determined for all bacteria tested in this study using the microdilution assay [85]. The inoculums of the bacterial strains were prepared from an overnight cultures and suspensions and were adjusted to 0.5 McF standard turbidity. The essential oils, dissolved in 5% dimethylsulfoxide (DMSO 5%), were first diluted to the highest concentration (100 mg/mL) to be tested. Then serial twofold dilutions were made in a concentrations range from 50 to 0.05 mg/mL in the 96-well plates were prepared by dispensing 100 µL aliquot from the stock solutions of each essential oil was added to 95 µL of the correspondent broth into the first well. Then, 100 µL from the serial dilutions were transferred into 10 consecutive wells. Finally, 5 µL of the inoculum of each microorganism was added to the wells. The last well containing 195 µL of nutrient broth without essential oil and 5 µL of the inoculum on each strip was used as the negative control. The final volume in each well was 200 µL. The plates were incubated at 37 °C for 18–24 h.

### 4.7. Statistical Analysis

All data from quantitative adhesion assays were expressed as Means ± Standard Deviation (S.D.). Each analysis was performed using the SPSS 16.0 Statistics Package for Windows. The differences in mean were calculated using the Duncan's multiple range tests for means with 95% confidence limit (*p* = 0.05).

## 5. Conclusions

The three essential oils tested in the present study possess different degrees of antibacterial activity against several Gram positive and Gram negative bacterial species isolated from fish and shellfish products. It seems that laurel essential oil possesses the highest antibacterial activity against all tested bacteria. Hence, they could potentially be used as a natural preservatives against food-borne pathogens, in order to inhibit lipid oxidation and delay microbial growth in fish and shellfish. These essential oils can also be mixed with fish diets to prevent the multiplication of natural spoilage bacteria. In rearing tanks of *Sparus aurata* or *Dicentrarchus labrax* fishes, or their live prey (rotifer cultures), these essential oils can reduce the count of the pathogenic bacteria like *Vibrio* spp. strains.

Additionally, the antioxidant-antiradical activity registered can be important to preserve marine products known to contain a number of components prone to chemical degradation. It seems that laurel essential oil was the most effective as compared to the dill and zinger oils. Therefore this oil can be used to prolong seafood shelf life because of its protective effects against both microbiological and chemical deterioration thus preventing off flavors and formation of all the above toxic agents.

Further experiments must be done to confirm the viability of the commercial use of these essential oils, and especially laurel volatile oil, if used to prevent the multiplication of natural spoilage fish and shellfish flora in the food industry, or in fish hatcheries to control the multiplication of pathogenic bacteria like *Vibrionaceae* and *Aeromonadaceae* families members in live prey cultures (*Artemia salina* and *Brachionus plicatilis*) and fish rearing tanks.

## Figures and Tables

**Table 1:** Biotypes obtained on Api strips, exoenzymes profile and adhesive properties of the 34 strains isolated from fish and shellfish products. (−): Negative; (+): Positive.

Samples	Code	Identification	Biotype on Api	% id	Exoenzymes Profile					Adhesive Properties	
					Haemolysis	Caseinase	DNase	Amylase	Lecithinase	Slime (CRA)	OD_595_ ± SD
***P. longirostris***	**R_58_**	***Klebsiella oxytoca***	5245573	93.6	γ	+	−	+	−	Red	0.28 ± 0.02
	**R_44_**	***Aeromonas hydrophila***	7772755	98.2	γ	+	+	+	+	Black	0.26 ± 0.06
	**R_63_**	***Vibrio alginolyticus***	7650745	82.2	β	+	+	+	−	Pink	0.21 ± 0.02
	**R_72_**	***Aeromonas hydrophila***	5652355	88.2	β	+	+	+	−	Bordeaux	0.41 ± 0.03
	**R_32_**	***Staphylococcus lugdunensis***	6314150	42.6	β	+	+	+	−	Pink	0.21 ± 0.09
	**R_53_**	***Staphylococcus sciuri***	6314050	72.5	β	+	+	+	−	Pink	0.19 ± 0.01
	**R_30_**	***Staphylococcus lentus***	6731771	99.6	β	+	+	+	−	Black	0.31 ± 0.04
	**R_64_**	***Staphylococcus xylosus***	6735753	99	β	+	+	+	−	Red	0.18 ± 0.02
***D. labrax***	**R_14_**	***Aeromonas hydrophila***	7576755	83.8	β	+	+	+	+	Bordeaux	0.27 ± 0.02
	**R_35_**	***Staphylococcus sciuri***	6314050	72.5	β	+	+	+	−	Pink	0.19 ± 0.04
	**R_3_**	***Aeromonas hydrophila***	5567745	82	γ	+	−	+	−	Red	0.22 ± 0.01
	**R_23_**	***Aeromonas hydrophila***	5777745	99.5	γ	+	−	+	−	Pink	0.21 ± 0.02
	**R_2_**	***Staphylococcus sciuri***	6334070	99	β	+	+	+	−	Red	0.28 ± 0.12
	**R_71_**	***Aeromonas hydrophila***	7535755	99.9	β	+	+	+	+	Pink	0.15 ± 0.01
	**R_16_**	***Enterobacter cloacae***	5245573	38.4	γ	+	−	+	−	Pink	0.26 ± 0.05
***M. galloprovincialis***	**R_5_**	***Aeromonas hydrophila***	7577754	99.9	β	+	−	+	+	Bordeaux	0.18 ± 0.04
	**R_29_**	***Aeromonas hydrophila***	7577776	99.9	β	+	+	+	−	Red	0.19 ± 0.03
	**R_70_**	***Aeromonas hydrophila***	7577755	99.3	β	+	−	+	+	Bordeaux	0.36 ± 0.09
	**R_47_**	***Aeromonas hydrophila***	7573744	99.8	β	+	+	+	+	Bordeaux	0.20 ± 0.01
	**R_22_**	***Aeromonas hydrophila***	5677254	99.2	β	+	+	+	−	Black	0.20 ± 0.01
	**R_13_**	***Aeromonas hydrophila***	7477754	99.9	β	+	+	+	−	Black	0.26 ± 0.01
***S. aurata***	**R_46_**	***Aeromonas hydrophila***	7777757	99.9	γ	+	−	+	−	Pink	0.19 ± 0.02
	**R_11_**	***Aeromonas hydrophila***	7777757	99.9	β	+	+	+	+	Red	0.16 ± 0.01
	**R_68_**	***Enterobacter cloacae***	5245573	38.4	γ	+	−	+	−	Pink	0.13 ± 0.01
	**R_40_**	***Staphylococcus sciuri***	6314050	72.5	β	+	+	+	−	Red	0.22 ± 0.05
	**R_9_**	***Klebsiella ornithinolytica***	5345573	91	γ	+	+	+	+	Pink	0.19 ± 0.01
	**R_4_**	***Aeromonas hydrophila***	7537745	99.6	β	+	−	+	+	Red	0.25 ± 0.10
	**R_36_**	***Staphylococcus sciuri***	6314050	72.5	β	+	+	+	−	Pink	0.27 ± 0.04
	**R_48_**	***Aeromonas hydrophila***	7577755	99.9	β	+	+	+	+	Bordeaux	0.20 ± 0.01
	**R_37_**	***Staphylococcus sciuri***	6314050	72.5	β	+	+	+	−	Pink	0.22 ± 0.05
	**R_18_**	***Aeromonas hydrophila***	7477777	78.1	β	+	+	+	+	Pink	0.19 ± 0.03
	**R_55_**	***Aeromonas hydrophila***	7777744	99.6	β	+	+	+	+	Black	0.30 ± 0.02
	**R_69_**	***Serratia odorifera***	7347573	95.2	γ	+	+	+	−	Red	0.37 ± 0.03
	**R_1_**	***Staphylococcus lugdunensis***	6314150	42.6	β	+	−	+	−	Red	0.18 ± 0.03

**Table 2:** Composition of *L. nobilis* (Ln), *A. graveolens* (Ag) and *Z. officinale* (Zo) essential oils. RI: Retention Index; Tr: Traces (less than 0.5%).

Constituents	RI	Essential Oils			Constituents	RI	Essential Oils		
		Ln	Ag	Zo			Ln	Ag	Zo
*(E)*-3-Hexen-1-ol	853	0.3			Cumin aldehyde	1241			0.2
2-Heptanone	891			Tr	Neral	1241		27.0	0.5
2-Heptanol	901			0.4	Carvone	1244		25.7	0.3
Tricyclene	928			0.3	Piperitone	1254			
α-Thujene	933	0.2	Tr		Geraniol	1256			0.7
α-Pinene	941	3.8	0.8	3.0	Geranial	1269			0.6
Camphene	955	0.5	0.1	11.5	Phellandral	1274			0.4
Sabinene	977	3.5	0.5		Bornyl acetate	1287	0.6		0.9
β-Pinene	982	3.6	0.2	0.2	2-Undecanone	1292			1.0
6-Methyl-5-hepten-2-one	987			1.0	α-Terpinyl acetate	1352	9.0		
Myrcene	993	0.3	1.5	1.0	α-Copaene	1377			0.2
δ-2-Carene	1003		0.6		Geranyl acetate	1383			0.8
α-Phellandrene	1006	Tr	1.4	2.0	β-Elemene	1392			Tr
δ-3-Carene	1013			Tr	Methyl eugenol	1403	3.6		
α-Terpinene	1020	0.3	0.1	1.4	β-Caryophyllene	1419	Tr		
*p*-Cymene	1027	0.5	0.7	1.2	*Allo*-Aromadendrene	1461			1.1
Limonene	1032	0.7	20.6		β-Chamigrene	1477			0.4
β-Phellandrene	1033			10.7	γ-Curcumene	1481			1.1
1.8-Cineole	1034	56.0		10.4	*ar*-Curcumene	1483			4.6
γ-Terpinene	1063	0.6	0.2	Tr	Valencene	1493			0.4
*cis*-Sabinene hydrate	1070	0.2	Tr		α-Zingiberene	1495			6.9
*cis*-Linalool oxide (furanoid)	1077	Tr		0.2	β-Bisabolene	1509			3.2
Terpinolene	1090	0.2	0.2		β-Sesquiphellandrene	1524			4.1
*p*-Cymenene	1091			0.7	Selina-3.7(11)-diene	1544			0.2
2-Nonanone	1092			0.4	Elemol	1549			0.2
Linalool	1101	3.8		1.9	*(E)*-Nerolidol	1565			0.5
*exo*-Fenchol	1118			0.4	Spathulenol	1577	0.4		
*cis-p*-Menth-2-en-1-ol	1123	Tr	0.1	0.3	Caryophyllene oxide	1582	0.3		
*trans*-Pinocarveol	1141	0.3			10-*epi*-γ-Eudesmol	1622			2.5
*trans-p*-Menth-2-en-1-ol	1142		0.1	0.2	Dill apiole	1623		8.0	
*cis*-Verbenol	1142	Tr			γ-Eudesmol	1631			0.8
Camphor	1145		4.4	0.5	β-Eudesmol	1650			0.8
Camphene hydrate	1150			0.8	α-Eudesmol	1653			0.5
Isoborneol	1158			0.5	α-Cadinol	1654			1.0
Borneol	1168	Tr		6.4	β-Bisabolol	1672			0.7
4-Terpineol	1178	5.2	0.3	1.0	α-Bisabolol	1684			0.2
*p*-Cymen-8-ol	1185			0.4	Monoterpene hydrocarbons		26.9	14.2	32.0
Dill ether	1186		0.6		Oxygenated monoterpenes		64.8	79.8	31.0
α-Terpineol	1190	4.7		3.2	Sesquiterpene hydrocarbons		0	0	22.2
Myrtenol	1195			0.2	Oxygenated sesquiterpenes		0	0.7	7.2
*cis*-Dihydrocarvone	1195		1.7		Phenylpropenoids		8.0	3.6	0
*trans*-Dihydrocarvone	1202		4.9		Others		0	0.3	2.8
*trans*-Piperitol	1207			0.2	Total identified		99.7	98.6	95.2

**Table 3:** Antioxidant activities of the three essential oils tested as compared to standard BHT, BHA and ascorbic acid.

Activities	Essential Oils			Standards		
	*L. nobilis*	*Z. officinale*	*A. graveolens*	BHT	Ascorbic Acid	BHA
DPPH (IC_50_; µg/mL)	135	470	3000	11.5		
RP (EC_50_; µg/mL)	1850	1900	2400		37.3	
β-carotenes (µg/mL)	3600	1900	4000			48
Ionsuperoxyde (O_2_^−^) (µg/mL)	620	320	400	1.5		

**Table 4:** Zone of growth inhibition (mean ± SD, mm), MICs and MBCs (mg/mL) caused by the three essential oils and by the standard antibiotics.

Species	Code	Essential Oils									Antibiotics	
		*L. nobilis*			*Z. officinale*			*A. graveolens*				
		ZI ± SD	MIC	MBC	ZI ± SD	MIC	MBC	ZI ± SD	MIC	MBC	1	2
***A. hydrophila***	**R_4_**	12.33 ± 0.58 ^hi^	0.05	>12.5	8.67 ± 0.58 ^ij^	0.05	>12.5	11.33 ± 0.58 ^ghi^	0.05	50	8	6
***A. hydrophila***	**R_47_**	20 ± 1 ^b^	0.05	25	13.67 ± 0.58 ^cd^	0.05	>0.78	15.33 ± 0.58 ^d^	0.05	>25	15	7
***A. hydrophila***	**R_48_**	22.33 ± 0.58 ^a^	0.10	50	14.33 ± 0.58 ^bc^	0.20	12.5	11.67 ± 0.58 ^gh^	0.10	50	10	6
***A. hydrophila***	**R_71_**	16.67 ± 0.58 ^c^	0.20	>25	13.67 ± 0.58 ^cd^	0.05	>25	13 ± 1 ^ef^	0.20	>12.5	15	6
***A. hydrophila***	**R_29_**	14.33 ± 0.58 ^efg^	0.10	>6.25	12.33 ± 0.58 ^efg^	0.10	>25	13 ± 0 ^ef^	0.10	50	6	20
***A. hydrophila***	**R_5_**	20.67 ± 0.58 ^b^	0.10	12.5	12.67 ± 0.58 ^ef^	0.10	12.5	25.33 ± 0.58 ^a^	0.10	50	11	8
***A. hydrophila***	**R_13_**	11.33 ± 0.58 ^j^	0.10	>12.5	7 ± 0 ^k^	0.05	>25	6 ± 0 ^l^	0.10	>12	13	6
***A. hydrophila***	**R_3_**	15.33 ± 0.58 ^d^	0.10	>25	15.33 ± 0.58 ^a^	0.10	>12.5	13.33 ± 0.58 ^e^	0.10	>12.5	19	6
***A. hydrophila***	**R_46_**	14.33 ± 0.58 ^def^	0.10	25	14.33 ± 0.58 ^bc^	0.05	>25	12.33 ± 0.58 ^efg^	0.10	>12.5	11	7
***A. hydrophila***	**R_22_**	11.67 ± 0.58 ^ij^	0.05	6.25	8.67 ± 0.58 ^ij^	0.05	12.5	7.33 ± 0.58 ^k^	0.10	>12.5	11	6
***A. hydrophila***	**R_14_**	13.67 ± 0.58 ^g^	0.05	>6.25	10.33 ± 0.58 ^h^	0.05	>25	8.67 ± 0.58 ^j^	0.05	>25	14	6
***A. hydrophila***	**R_70_**	11.67 ± 0.58 ^ij^	0.20	>25	11.67 ± 0.58 ^fg^	0.10	50	11.33 ± 0.58 ^ghi^	0.05	>12.5	7	6
***A. hydrophila***	**R_11_**	14.67 ± 0.58 ^de^	0.10	>25	12.67 ± 0.58 ^ef^	0.05	>6.25	14.67 ± 0.58 ^d^	0.05	>12.5	10	7
***A. hydrophila***	**R_55_**	12 ± 1 ^ij^	0.05	12.5	12.00 ± 0 ^fg^	0.05	>3.13	12 ± 1 ^fgh^	0.10	12.5	10	6
***A. hydrophila***	**R_18_**	13.67 ± 0.58 ^efg^	0.05	12.5	12.67 ± 0.58 ^ef^	0.05	50	11 ± 0 ^hi^	0.05	>25	11	7
***A. hydrophila***	**R_23_**	14.67 ± 0.58 ^de^	0.05	>6.25	12.33 ± 0.58 ^efg^	0.05	50	13 ± 0 ^ef^	0.10	>25	12	6
***A. hydrophila***	**R_44_**	13.33 ± 0.58 ^fg^	0.10	>25	6 ± 0 ^l^	0.05	50	6 ± 0 ^l^	0.05	>12.5	12	6
***A. hydrophila***	**R_72_**	15.33 ± 0.58 ^d^	0.05	12.5	12.33 ± 0.58 ^efg^	0.05	>0.78	12.33 ± 0.58 ^efg^	0.05	>25	11	6
***E. cloacae***	**R_68_**	10.33 ± 0.58 ^k^	0.20	>25	9 ± 0 ^i^	0.05	>25	11.67 ± 0.58 ^gh^	0.05	>25	8	8
***E. cloacae***	**R_16_**	11.67 ± 0.58 ^ij^	0.05	>50	8 ± 0 ^j^	0.10	>25	7.67 ± 0.58 ^k^	0.10	50	12	7
***K. ornithinolytica***	**R_9_**	17 ± 0 ^c^	0.05	6.25	11.33 ± 0.58 ^g^	0.20	>25	8.67 ± 0.58 ^j^	0.05	>25	15	7
***K. oxytoca***	**R_58_**	14.67 ± 0.58 ^de^	0.05	>12.5	13 ± 0 ^de^	0.05	>12.5	12 ± 0 ^fgh^	0.10	50	10	11
***S. lentus***	**R_30_**	6 ± 0 ^l^	0.05	>6.25	6 ± 0 ^l^	0.05	>3.13	11.33 ± 0.58 ^ghi^	0.05	>25	22	6
***S. lugdunensis***	**R_1_**	14.33 ± 0.58 ^def^	0.10	>25	10.33 ± 0.58 ^h^	0.05	>12.5	10.33 ± 0.58 ^i^	0.10	>25	19	6
***S. lugdunensis***	**R_32_**	15.33 ± 0.58 ^d^	0.05	>12.5	14.67 ± 0.58 ^ab^	0.20	>12.5	17.67 ± 0.58 ^c^	0.39	50	22	20
***S. odorifera***	**R_69_**	14.33 ± 0.58 ^def^	0.10	>12.5	11.33 ± 0.58 ^g^	0.05	>12.5	11.33 ± 0.58 ^ghi^	0.10	>12.5	8	7
***S. sciuri***	**R_40_**	14.67 ± 0.58 ^de^	0.05	>25	11.33 ± 0.58 ^g^	0.10	>25	11.33 ± 0.58 ^ghi^	0.10	50	15	7
***S. sciuri***	**R_35_**	14.67 ± 0.58 ^de^	0.05	>25	11.67 ± 0.58 ^fg^	0.05	>25	13.33 ± 0.58 ^e^	0.10	>25	24	6
***S. sciuri***	**R_36_**	14.33 ± 0.58 ^def^	0.05	>6.25	12.33 ± 0.58 ^efg^	0.20	>6.25	14.67 ± 0.58 ^d^	0.05	>25	10	13
***S. sciuri***	**R_37_**	14.67 ± 0.58 ^de^	0.05	>25	8.67 ± 0.58 ^ij^	0.05	>6.25	11.67 ± 0.58 ^gh^	0.10	>25	7	15
***S. sciuri***	**R_2_**	14 ± 1 ^ef^	0.05	>12.5	11.67 ± 0.58 ^fg^	0.10	>12.5	11.67 ± 0.58 ^gh^	0.10	50	8	20
***S. sciuri***	**R_53_**	14.67 ± 0.58 ^de^	0.05	12.5	13.67 ± 0.58 ^cd^	0.05	1.56	20 ± 1 ^b^	0.05	>12.5	10	7
***S. xylosus***	**R_64_**	14.33 ± 0.58 ^def^	0.05	>6.25	12.33 ± 0.58 ^efg^	0.05	>12.5	15.33 ± 0.58 ^d^	0.10	>12.5	12	8
***V. alginolyticus***	**R_63_**	13 ± 0 ^gh^	0.05	>6.25	10.33 ± 0.58 ^h^	0.05	>25	12.33 ± 0.58 ^efg^	0.10	50	8	7

**MIC**: Minimum Inhibitory Concentration (mg/mL). **MBC**: Minimum Bactericidal Concentration (mg/mL). **IZ**: Inhibition zone in diameter (mm ± SD) around the discs impregnated with 10 mg/disk of essential oil including disc diameter. **SD**: standard deviation; (a–l): Means followed by the same letters are not significantly different at *p* = 0.05 based on Duncan’s multiple range tests. **1**: Erythromycin (15 µg). **2**: Kanamycin (30 µg).
